# Engaging the Innate and Adaptive Antitumor Immune Response in Lymphoma

**DOI:** 10.3390/ijms22073302

**Published:** 2021-03-24

**Authors:** Clifford M. Csizmar, Stephen M. Ansell

**Affiliations:** 1 Department of Internal Medicine, Mayo Clinic, Rochester, MN 55905, USA; csizmar.clifford@mayo.edu; 2 Division of Hematology, Mayo Clinic, Rochester, MN 55905, USA

**Keywords:** non-Hodgkin lymphoma, Hodgkin lymphoma, tumor microenvironment, immunotherapy, immune checkpoint inhibitors, bispecific antibodies, BiTEs

## Abstract

Immunotherapy has emerged as a powerful therapeutic strategy for many malignancies, including lymphoma. As in solid tumors, early clinical trials have revealed that immunotherapy is not equally efficacious across all lymphoma subtypes. For example, immune checkpoint inhibition has a higher overall response rate and leads to more durable outcomes in Hodgkin lymphomas compared to non-Hodgkin lymphomas. These observations, combined with a growing understanding of tumor biology, have implicated the tumor microenvironment as a major determinant of treatment response and prognosis. Interactions between lymphoma cells and their microenvironment facilitate several mechanisms that impair the antitumor immune response, including loss of major histocompatibility complexes, expression of immunosuppressive ligands, secretion of immunosuppressive cytokines, and the recruitment, expansion, and skewing of suppressive cell populations. Accordingly, treatments to overcome these barriers are being rapidly developed and translated into clinical trials. This review will discuss the mechanisms of immune evasion, current avenues for optimizing the antitumor immune response, clinical successes and failures of lymphoma immunotherapy, and outstanding hurdles that remain to be addressed.

## 1. Introduction

Immune therapies have emerged as powerful treatment modalities for many solid and hematologic malignancies. Tumor-targeting antibodies have remained a cornerstone of therapeutic regimens after the introduction of rituximab at the turn of the century, and the discovery of checkpoint inhibitor ligands has enabled the development of antibodies capable of modulating the antitumor immune response [1]. Checkpoint inhibitor therapy has since demonstrated marked efficacy in select solid tumors [2,3,4]. However, early clinical data quickly revealed large discrepancies in the response rates of different lymphoma subtypes, with classical Hodgkin lymphoma (cHL) responding much more favorably to checkpoint inhibition compared to most B-cell non-Hodgkin lymphomas (B-NHLs) [5]. These observations, combined with a growing understanding of lymphoma biology, have implicated the tumor microenvironment (TME) as a major determinant of treatment response and prognosis.

As a malignancy arising from cells of the immune system, lymphoma cells interact with their microenvironment in unique ways that contribute to immune evasion [6]. In particular, cytokine signaling and chemokine secretion recruit and expand immunosuppressive cell populations within the TME. Moreover, the loss of major histocompatibility complexes and the expression of immunosuppressive ligands further contribute to the impairment of both innate and adaptive effector cells. These behaviors allow lymphoma cells to curate an environment that suppresses cytotoxic immune cell activity and rather promotes and sustains malignant cell proliferation.

Accordingly, agents rationally designed to overcome these barriers are being rapidly developed and translated into clinical trials. This review will discuss how the TME contributes to immune evasion in lymphoma, contemporary options for eliciting an antitumor immune response, the clinical outcomes of lymphoma immunotherapy, and the remaining challenges that have yet to be addressed.

## 2. The Tumor Microenvironment in Lymphoma

**Figure 1:**
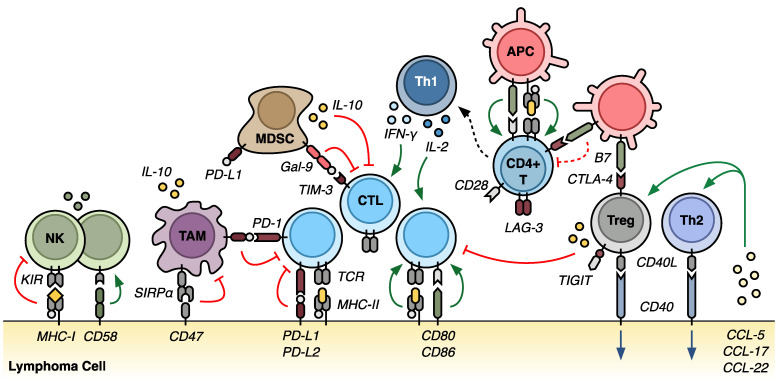
The tumor microenvironment in lymphoma. Numerous inhibitory and stimulatory interactions shape the lymphoma microenvironment. Activation of CD8+ cytotoxic T-lymphocytes (CTLs) requires two steps, including T-cell receptor (TCR) recognition of a peptide antigen in the context of major histocompatibility complex (MHC) and costimulation between CD28 and a B7 protein family member (CD80 or CD86). CTL proliferation and activity can be further supported by interferon gamma (IFN-γ) and interleukin 2 (IL-2) secretion, largely by proinflammatory CD4+ T-helper 1 (Th1) cells. Antigen-presenting cells (APCs) can also present MHC-restricted epitopes to CD4+ T-cells; however, T-cell expression of cytotoxic T-lymphocyte antigen 4 (CTLA-4) competes with costimulatory CD28 to dampen T-cell activation. The protein product of lymphocyte activation gene 3 (LAG-3) similarly competes for binding to MHC-II. Multiple T-cell populations—including regulatory T-cells (Tregs)—express T-cell immunoreceptor with immunoglobulin and ITIM domains (TIGIT), which both reduces IFN-γ secretion and promotes immunosuppressive interleukin (IL)-10 release. This cytokine signaling, combined with the secretion of various chemokine ligands (CCL-5, 17, and 22) by the lymphoma cells, recruits Th2 cells, myeloid-derived suppressor cells (MDSCs), and tumor-associated macrophages (TAMs) to the environment. The Th2 cells support lymphoma cell growth by means of interactions between CD40 and the CD40 ligand (CD40L). MDSCs express galectin-9 (Gal-9), which binds to T-cell immunoglobulin and mucin domain 3 (TIM-3) to drive T-cell exhaustion. TAMs, MDSCs, and some lymphoma subtypes also express programmed cell death ligands 1 and 2 (PD-L1 and PD-L2), which further impair T-cell function and, ultimately, lead to T-cell apoptosis. Lymphoma cells further shield themselves from the innate immune system via the expression of CD47, which binds to signal regulatory protein alpha (SIRPα) on TAMs to inhibit phagocytosis. MHC-mediated interactions with killer cell immunoglobulin-like receptor (KIR) on NK cells provide additional self-tolerance against NK-mediated cytotoxicity. Collectively, these various signals and interactions create an environment of immune dysfunction that sustains and promotes malignant growth.

The TME is a heterogeneous milieu of mesenchymal stromal cells, immune cells, tumor cells, and associated cytokines [7,8]. It plays pivotal roles in supporting tumor growth, regulating immune surveillance of the malignant cells, and facilitating subsequent immune evasion (Figure 1) [9]. As these complex processes often culminate in acquired drug resistance, the TME has become a research focus that spans the drug development pipeline [10,11]. This section discusses the putative mechanisms by which the TME contributes to immune evasion and, ultimately, therapeutic failures in lymphoma and its subtypes.

### 2.1. Overview of the Tumor Microenvironment

The lymphoma TME is comprised of diverse cells that, depending on the disease subtype, are present in varying abundances and proportions [6,9,12]. T-lymphocytes—including CD4+ T-helper cells (Ths), CD4+/FOXP3+ regulatory T-cells (Tregs), and CD8+ cytotoxic T-lymphocytes (CTLs)—are fundamental for immune surveillance and disease progression and, thus, continue to be studied extensively in essentially all lymphoma subtypes [9,13]. In general, Th2 cells tend to support tumor cell growth through CD40–CD40 ligand (CD40L) interactions [14,15]. Meanwhile, Th1 cells promote inflammation via secretion of interleukin (IL)-2, IL-12, and interferon (IFN)-γ, leading to the activation of CTLs, antigen-presenting cells (APCs), and natural killer (NK) cells [16]. This inflammatory response is tempered by Tregs via a multitude of mechanisms, including the expression of cytotoxic T-lymphocyte antigen 4 (CTLA-4) and the secretion of IL-10, which serve to inhibit CTL function. Moreover, programmed cell death protein 1 (PD-1) is expressed on both activated CD4+ and CD8+ T-cells and, upon interaction with its ligands (PD-L1 and PD-L2), suppresses T-cell activity and induces exhaustion and anergy [17].

Other lymphoid cells commonly found in the TME include nonmalignant B-cells and NK cells. While nonmalignant B cells are frequently encountered in the TME of indolent lymphomas, their role in disease maintenance and progression remains poorly defined [8,9]. NK cells are present in varying quantities, recruited by IL-2 and IFN-γ, as above, but are almost universally functionally deficient [18,19,20]. Several mechanisms have been implicated in this NK cell dysfunction, and these are discussed below.

Myeloid cells are also abundant within the lymphoma TME. Tumor-associated macrophages (TAMs) were traditionally identified by expression of the pan-macrophage marker CD68, although more recent studies have revealed CD163 expression as a defining feature of M2-polarized macrophages with prognostic value [21]. TAMs promote tumor growth by stimulating angiogenesis as well as tumor cell migration and invasion [22]. They also suppress the antitumor immune response through the expression of PD-L1 and the secretion of IL-10 [23,24,25]. Moreover, the presence of CD163+ TAMs has long been associated with poor clinical outcomes in a variety of malignancies, including lymphoma [26,27,28]. Myeloid-derived suppressor cells (MDSCs) are a heterogeneous population of immature monocytes and granulocytes [29]. MDSCs exert immune suppressive effects through the expression of galectin-9 (Gal-9), which interacts with T-cell immunoglobulin and mucin domain 3 (TIM-3) on T-cells to impair the lymphoid immune response [30,31]. MDSCs also produce nitric oxide, secrete IL-10, and express PD-L1, further dampening immune function [24,32]. Similar to TAMs, increased frequencies of MDSCs are correlated with more aggressive disease in both Hodgkin and non-Hodgkin lymphomas [33,34]. Eosinophils and mast cells expressing CD30 ligand are prominent in classical Hodgkin lymphoma (cHL), where they portend a worse prognosis [35,36,37].

These cells interact extensively with the various stromal cells and extracellular matrix of the local environment; the latter is comprised largely of collagen and reticular fibers. In cHL, for example, fibroblasts secrete proinflammatory cytokines, growth factors, and pro-angiogenic factors that promote tumor survival and mitigate Hodgkin and Reed-Sternberg (HRS) cell apoptosis [38,39]. In follicular lymphoma (FL), fibroblastic reticular cells secrete stromal-derived growth factor 1 (SDF-1, also known as CXCL12), which stimulates follicular T-helper cells (TFHs) that support malignant cell growth through CD40–CD40L interactions [40,41]. Moreover, the survival of neoplastic cells in mucosa-associated lymphoid (MALT) lymphoma and mantle cell lymphoma (MCL) is heavily dependent on the presence of stromal cells, especially in chemotherapy-resistant settings [42,43,44].

### 2.2. Inflamed and Noninflamed Lymphomas

The degree of T-cell infiltration into the lymphoma TME has garnered the broad classification of “inflamed” and “noninflamed” lymphoma subtypes [45,46,47]. T-cell-inflamed tumors are characterized by robust immune cell infiltration (prominently T-cells) [6], upregulation of T-cell activation gene signatures [48,49], alterations that facilitate immune escape [50,51,52], and aberrant NF-κB activation [53,54]. Collectively, these features generally confer sensitivity to immune checkpoint inhibitor (ICI) therapy [55]. In contrast, T-cell-noninflamed tumors have a paucity of infiltrating immune cells, few genetic immune escape mechanisms, and are often less susceptible to ICIs [56].

Classical Hodgkin lymphoma is a prototypical inflamed lymphoma, with robust recruitment of infiltrating immune cells such that they vastly outnumber the malignant HRS cells. HRS cells secrete numerous chemokines, including CCL5, CCL17, and CCL22, that recruit Th and Treg cells to the TME [13]. Initial studies suggested that Th2 cells were the dominant T-cell infiltrate, as HRS cell survival appears dependent on Th2 cell stimulation [57,58,59] via CD40–CD40L interactions that promote aberrant NF-κB pathway activation [60,61,62]. More recent studies, however, have demonstrated that proinflammatory Th1 cells are more heavily enriched in the cHL TME than the supportive Th2 cells, with a concomitant abundance of Th1 transcription factors (T-BET) and cytokines (IFN-γ) [63,64]. This suggests that while an inflammatory immune response against the HRS cells is mounted, it is subverted by the HRS cells and thus ineffective (as discussed below). This immune evasion is enhanced by the abundance of immunosuppressive Tregs that are also recruited to the TME, where they facilitate exhaustion of the effector T-cells [63,65]. The abundance of immune infiltrates in cHL likely underpins the clinical efficacy of ICI therapy in relapsed/refractory disease [66,67], as checkpoint blockade helps alleviate effector cell exhaustion and facilitates reactivation of antitumor T-cell responses [68,69]. Likewise, primary mediastinal B-cell lymphoma (PMBCL), primary central nervous system lymphoma (PCNSL), gray zone lymphoma (GZL), and primary testicular lymphoma (PTL) are thought to have T-cell-inflamed microenvironments, with upregulation of PD-L1/PD-L2, and favorable clinical responses to ICI therapy have been observed in these diseases [70,71,72,73,74,75].

On the contrary, many lymphomas have a noninflamed microenvironment, including chronic lymphocytic leukemia (CLL), Burkitt lymphoma (BL), and most diffuse large B-cell lymphomas (DLBCLs). The lack of CTL infiltrate in these malignancies may be augmented by an inherently high proliferation rate that excludes immune infiltration or molecular expression profiles that dampen the immune response [76,77,78,79]. In aggressive B-cell lymphomas such as BL and DLBCL, the normal lymph node architecture is effaced by the rapidly dividing malignant cells, which physically impede T-lymphocyte infiltration. This behavior is enabled, in part, by gene rearrangements in *MYC*, *BCL2*, and/or *BCL6*, which impart strong autonomous cell proliferation signals and, thereby, alleviate the tumor cells’ dependence on the microenvironment [76,80]. Moreover, high-grade B-cell lymphomas are often enriched in *EZH2*-activating mutations that ultimately serve to downregulate human leukocyte antigen (HLA) expression [81]. Furthermore, DLBCLs arising from germinal center B-cells (GCBs) typically lack genomic amplification and copy gain of *PD-L1*, and, thus, PD-L1 overexpression is rare in these settings [48,50]. Collectively, these features have contributed to disappointing outcomes in trials of ICI in DLBCL [82,83].

Follicular lymphoma also arises from GCBs, but with a more indolent course; it does not proliferate rapidly enough to efface the TME in the same manner as high-grade lymphomas. Rather, FL reprograms the surrounding environment to partly resemble a normal follicle that can support tumor cell growth through sustained, albeit inappropriate, activation of the B-cell receptor (BCR) [84,85,86]. There is a substantial secretion of IL-4, which recruits TFH, follicular dendritic cells (FDCs), and Tregs that promote tumor growth through BCR signaling and CD40–CD40L interactions [78,79]. The IL-4 also stimulates macrophages and stromal cells to secrete SDF-1/CXCL12, which helps polarize CXCR4+ (the receptor for SDF-1/CXCL12) TFHs in a manner similar to a developing follicle [40,41]. As such, the FL cells seem to recruit, polarize, and maintain their own TME in a manner that promotes tumor survival and ignores immune surveillance [6,9]. In that regard, despite a high number of PD-1+ tumor-infiltrating lymphocytes (TILs), PD-1 expression has been correlated with variable outcomes in FL, ranging from improved prognosis to more rapid progression or high-grade transformation [87,88,89]. Likewise, ICI trials in FL have yielded only modest results [90].

### 2.3. Requisites for an Adequate Antitumor Immune Response

Under normal circumstances, immune system function is closely regulated to carefully balance efficient destruction of foreign pathogens with preservation of host tissues. Failure to mount a sufficient immune response to an antigen leaves the host susceptible to increased frequency and severity of infection. Conversely, overzealous responses to otherwise innocuous- or self-antigens manifest as a spectrum of autoimmune disorders. In lymphoma, as in most malignancies, immune system dysfunction contributes to disease progression and relapse.

**Figure 2:**
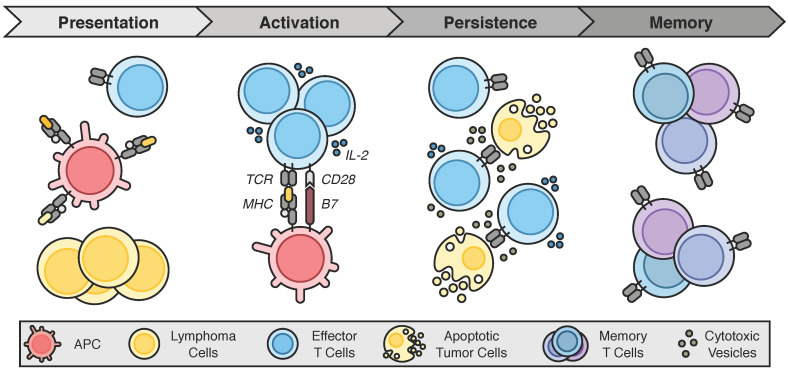
An adequate immune response in lymphoma. An appropriate antitumor immune response requires four fundamental steps. First, tumor antigens must be recognized as foreign via presentation in the context of major histocompatibility complex (MHC) domains. Presentation is classically accomplished by an antigen-presenting cell (APC), such as a dendritic cell or a macrophage. Following recognition, the second step is the activation and expansion of immune effector cells. In the case of T-cells, this requires costimulation and sufficient cytokine support. Third, the effector cell populations must persist in sufficient quantities and maintain activity until the malignant cell cohort is eliminated in its entirety. Ideally, the immune response would culminate in immunologic memory capable of swiftly responding to future tumor antigen encounters. Unfortunately, lymphomas have evolved a plethora of mechanisms to subvert an effective immune response.

In cancer, an optimal immune response requires four broad processes (Figure 2) [10]. First, tumor-associated antigens (TAAs) must be recognized as foreign. This requires that the antigen be classically presented in the context of major histocompatibility complex (MHC) domains by an APC such as a dendritic cell or a macrophage. In lymphomas, the malignant B- or T-cells can act as APCs, albeit very inefficiently, due to reduced surface MHC expression [91]. Moreover, a fundamental issue in cancer is that the “pathogen” is host tissue that expresses primarily—if not exclusively—self-antigens. Thus, tumors with low mutational burdens and minimal neoantigen expression are poorly immunogenic [92].

Once a “foreign” neoantigen has been recognized, the second requisite step is the activation and expansion of immune effector cells. In the case of T-cells, this activation itself is a two-step process, requiring two distinct stimulatory signals [93]. First, the T-cell receptor (TCR) must recognize a tumor antigen presented on the cell surface in the context of MHC molecules. A second costimulatory signal must then be received via the engagement of B7-1 (CD80) or B7-2 (CD86) molecules on the APC and CD28 on the T-cell. Receipt of both signals triggers T-cell priming and proliferation, which are further enhanced through the secretion of IL-2 [93]. Notably, other costimulatory signals have since been identified, including CD134 (OX40), CD137 (4-1BB), and CD27 [94]. In the absence of a second costimulatory signal, however, the T-cells become anergic [95]. This process of T-cell activation is further regulated by both central and peripheral checkpoints [17]. The central checkpoint occurs during the priming of naïve T-cells in the lymphoid organ, where antigen stimulation upregulates CTLA-4 expression on the T-cell. CTLA-4 then competes with CD28 for binding to B7, halting the T-cell activation process and yielding tolerance/anergy [96]. In the periphery, PD-L1 and PD-L2 expressed on target cells binds to PD-1 on CTLs. PD-1 then recruits the protein tyrosine phosphatase SHP2 (Src homology-2 domain-containing protein tyrosine phosphatase-2) to the TCR complex, resulting in dephosphorylation and attenuation of TCR signaling [66] and, thus, T-cell exhaustion.

Third, the expanded effector cell populations must persist—both in sufficient quantity and activity—until the entirety of the malignant cell cohort is eliminated. T-cell persistence is multifactorial and heavily influenced by the balance of receptor signaling, cytokine stimulation, and memory T-cell differentiation [97,98]. Finally, an ideal immune response is one that generates immunologic memory so that future encounters with the TAA can be recognized and eliminated swiftly before creating complications for the patient. This is typically accomplished via the formation of various memory T-cell subsets, including central, resident, and effector memory T-cell populations [99,100].

Unfortunately, as is discussed in the next section, malignancies have evolved numerous mechanisms by which to subvert these requisite steps, thereby preventing the immune system from mounting an adequate antitumor response.

## 3. Mechanisms of Immune Evasion

Clinical successes and failures have highlighted the importance of the TME in determining the response to therapy and disease prognosis. They have also motivated the rapid discovery and elucidation of a plethora of mechanisms that contribute to cancer’s ability to evade and subvert immune surveillance. In lymphoma, these mechanisms span both the innate and adaptive immune systems and are discussed below.

### 3.1. Loss of Major Histocompatibility Complexes

A primary mechanism by which both Hodgkin and non-Hodgkin lymphomas evade the immune system is through the loss of MHC class I and class II molecules, which reduces the presentation of TAAs to the immune system. Immunohistochemistry (IHC) analysis of biopsy samples from 108 patients with cHL demonstrated decreased expression of MHC class I and II molecules in 79% and 67% of cases, respectively [101]. Likewise, aberrancies in MHC expression have been seen in DLBCL (62%), PCNSL (77%), and testicular lymphomas (87%) [91]. In most cases, decreases in MHC expression are associated with inferior clinical outcomes, including shorter durations of progression-free survival (PFS) and reduced overall survival (OS) [101,102,103].

Multiple genetic alterations have been implicated in altered MHC expression. Mutations and deletions within the *B2M* gene yield loss-of-function of β2-microglobulin (β2M), thus preventing the surface expression of MHC class I [104]. Such deficits have been observed in up to 70% of cHL cases and 29% of DLBCL cases [104,105]. Similarly, genomic breaks in the class II transactivator gene (*CIITA*) are associated with reduced expression of MHC class II in DLBCL, PMBCL, and cHL [106,107]. Finally, HLA gene deletions and recombinations in chromosome 6p21.32 are highly prevalent (60–72%) in extranodal large cell lymphomas (PCNSL and PTL) and are infrequently seen (0–29%) in nodal large cell lymphomas [75,108].

### 3.2. Expression of Immunosuppressive Ligands

The upregulation of immunosuppressive ligands within the TME has become perhaps the most heavily investigated mechanism of immune evasion as it underpins the clinical efficacy of ICIs. As such, many stimulatory and inhibitory ligands have been identified and evaluated as therapeutic targets in lymphoma [11]. While not an exhaustive list, major targets include the PD-1/PD-L1/PD-L2 axis, CTLA-4, TIM-3, lymphocyte activation gene 3 (LAG-3), and T-cell immunoreceptor with immunoglobulin and ITIM domains (TIGIT) (Figure 1).

PD-L1 (CD274) and PD-L2 (PDCD1LG2 or CD273) are variably expressed by malignant lymphoma cells and are the cognate ligands for PD-1 expressed on CTLs. PD-L1/2 signal through PD-1 to inhibit T-cell function, promote exhaustion, and ultimately drive T-cell apoptosis [17]. Amongst lymphomas, PD-L1 overexpression is most pronounced in cHL, underpinning the success of ICI in this setting [109]. This overexpression is often driven by genetic alterations—primarily copy-number alterations—in chromosome 9p24.1 that lead to *PD-L1* and *PD-L2* gene amplification [12,110]. *JAK2* is also contained within the 9p24.1 amplicon, and Janus kinase 2 (JAK2) overexpression augments JAK/STAT (signal transducer and activator of transcription) signaling and further augments PD-L1 expression [110]. Similar mutational landscapes have been seen in PMBCL, PCNSL, PTL, and GZL, which may explain the clinical responses to ICI in these NHL subtypes [73,74,75]. In contrast, PD-L1 upregulation is much less frequent in other DLBCLs, occurring in approximately 11–25% of cases [48,50,111]. Otherwise, chronic viral infection with Epstein-Barr virus (EBV) has also been shown to promote PD-L1 and PD-L2 overexpression through activation of the activator protein-1 (AP-1) transcription factor [112]. Finally, PD-L1 and PD-L2 are expressed on other cells within the TME, such as TAMs and MDSCs, promoted by local IFNγ and IL-10 secretion [24,49]. In DLBCL, this PD-L1 expression on TAMs has been associated with a poor clinical prognosis [113].

CTLA-4 is expressed on Tregs, CD4+, and CD8+ T-cells, where it serves as a negative regulator of T-cell activation [96]. CTLA-4 acts as an alternate receptor for the otherwise costimulatory B7 molecules (CD80 and CD86). When the B7 molecules bind to CTLA-4 instead of CD28, the “second signal” required for T-cell activation is prevented, driving the T-cell into anergy instead. CTLA-4 is a critical mechanism by which Tregs contribute to an immunosuppressive environment [114], and it has been shown to be a crucial target in solid malignancies. Unfortunately, CTLA-4 inhibition has been less successful in lymphomas [82].

TIM-3 was originally identified on Th1 cells [115] but has since been shown to be expressed broadly throughout the TME, including on activated CD8+ T-cells, Tregs, NK cells, monocytes, macrophages, and dendritic cells [116]. The cognate ligand of TIM-3, Gal-9, is expressed by some lymphoma cells and MDSCs, where it serves to drive Th1 cell death and CTL immune exhaustion and, thus, impair antitumor T-cell responses [117]. Indeed, TIM-3 overexpression and exhaustion of TIM-3+ TILs have been shown to correlate with inferior outcomes in DLBCL [34,118]. The precise mechanisms by which TIM-3 expression on innate immune cells (i.e., NK cells, macrophages, and dendritic cells) contributes to immune dysfunction remain to be elucidated [116].

LAG-3 (CD223) is also broadly expressed on activated CD4+ and CD8+ T-cells, Tregs, dendritic cells, and a subset of NK cells [119,120]. It is structurally similar to the CD4 coreceptor and similarly binds to MHC class II, although the additional binding partners C-type lectin domain family 4 member G (CLEC4G, or LSECtin) and galectin-3 have been identified more recently [121,122,123]. After TCR engagement, LAG-3 associates with CD3 to dampen signal transduction and mitigate T-cell activation, although the precise mechanisms remain unclear [124]. On Treg cells, LAG-3 expression correlates with IL-10 secretion, although the direct contribution to Treg-mediated immune suppression is still under investigation [120,125].

TIGIT is expressed on NK cells and several T-cell populations, including activated, memory, follicular helper, and regulatory T-cells [116,126]. It is a coinhibitory member of the CD28 family and binds to the poliovirus receptor (CD155) on APCs and tumor cells [127]. Signaling through TIGIT contributes to reduced NK cell degranulation and cytotoxicity [128,129], reduced T-cell activation and INFγ secretion [127,130], and secretion of IL-10 by Treg cells [131]. Ultimately, antibodies against TIM-3, LAG-3, and TIGIT are all under investigation for the treatment of various solid and hematologic malignancies.

### 3.3. Recruitment and Expansion of Immunosuppressive Cell Populations

Dynamic recruitment and expansion of immunosuppressive cells play a fundamental role in the TME, and much has been written about this topic [6]. In lymphoma, several cell populations have emerged as especially important regulators of immune function—including Tregs, TFHs, MDSCs, and TAMs (Figure 1). As previously discussed, FOXP3+ Tregs are recruited to the TME through chemokine secretion by the lymphoma cells (including CCL5, CCL17, and CCL22) [13]. Transforming growth factor β (TGF-β) secretion also promotes the differentiation of naïve T-cells into Tregs [132]. The Tregs secrete IL-10 and express CTLA-4 and LAG-3, all of which serve to dampen the antitumor response of CTLs. Moreover, Tregs express CD40L and can promote the growth of cHL cells through stimulatory interactions with CD40, propagating the cycle of chemokine secretion, Treg recruitment, and effector T-cell suppression. Similarly, FL cells secrete IL-4 that recruits Tregs and TFH cells [78,79]. Again, CD40–CD40L interactions with the TFH cells strongly promote FL survival.

Granulocytic and monocytic MDSCs have been identified in varying quantities across numerous lymphoma subtypes, including cHL, DLBCL, and indolent NHLs [32,33]. They are induced and expanded by a number of soluble factors produced by the tumor and stroma, including vascular endothelial growth factor (VEGF), granulocyte-macrophage colony-stimulating factor (GM-CSF), IL-4, IL-6, and IL-10 [133]. Their roles in the TME are likely multifactorial and still under investigation. However, they have been shown to be a predominant producer of Gal-9, which inhibits TIM-3+ effector T-cells [30,31]. They also secrete IL-10 and express PD-L1, further hampering antitumor immune activity [32]. Indeed, increased numbers of MDSCs have been correlated with more aggressive disease courses in both cHL and NHL [33,34].

Likewise, TAMs have been repeatedly shown to contribute to poor outcomes in both solid and hematologic malignancies. Early work demonstrated that TAM gene expression signatures were enriched in cHL samples after primary treatment failure and that increased numbers of TAMs in the cHL environment were associated with both higher rates of disease relapse and shorter OS after autologous stem cell transplant [26]. The cHL microenvironment is also enriched with PD-L1+ TAMs, congregating around both HRS cells and PD-1+/CD4+ T-cells, suggesting that TAMs may shield the HRS cells from T-cell-mediated lysis and/or drive T-cell dysfunction, respectively [27]. Lymphoma cells shield themselves from TAM phagocytosis via the upregulation of CD47 [134]. CD47 binds to signal regulatory protein alpha (SIRPα) on TAMs and triggers a signaling cascade that inhibits the recruitment of myosin to the phagocytic interface, thereby suppressing phagocytosis [135]. Higher *CD47* mRNA transcripts have been seen in primary DLBCL samples from patients refractory to chemoimmunotherapy, and blockade of the CD47-SIRPα interaction enhances the phagocytosis of various NHL cell lines in vitro [136]. These findings (and others) have supported the clinical investigation of anti-CD47 antibodies in NHL, with promising early results [137].

### 3.4. Secretion of Exhaustive and Suppressive Cytokines

Immune evasion in lymphoma is facilitated by a rich and complex milieu of cytokines, chemokines, and soluble factors. For example, many cells in the TME secrete IL-10, which broadly inhibits Th1 and CTL function and recruits MDSCs (which themselves secrete IL-10) [24,65]. FL cells and their associated TFHs secrete an abundance of IL-4 that recruits, polarizes, and maintains the dysfunctional follicle microenvironment [40,78]. FDCs further enhance cell trafficking throughout the follicle through overexpression of CXCL12 and CXCL13, which facilitate the migration of CXCR4+ and CXCR5+ TFHs, respectively [41,138]. Moreover, many B-cell lymphomas secrete TGF-β to skew the differentiation of CD4+ T-cells towards Tregs [132]. Although normally associated with T-cell activation, prolonged secretion of IL-12 within the TME contributes to the upregulation of TIM-3 and T-cell exhaustion [139]. Finally, the presence of soluble PD-L1 correlates with poor prognosis in DLBCL [140,141].

### 3.5. Low Tumor Mutational Burden

Experience with ICI therapy in solid tumors has demonstrated a clear correlation between tumor mutational burden (TMB) and treatment efficacy [142]. This response is attributed to the increased expression of tumor neoantigens that are distinct enough from “self” peptides to permit an antitumor immune response. Unfortunately, the TMB of most B-cell lymphomas is significantly lower than in solid tumors sensitive to ICI treatment (e.g., melanoma, lung, and colorectal) [143]. Exceptions include PMBCL and cHL, which have been recently shown to have relatively high TMB, exhibit microsatellite instability (MSI), and feature apolipoprotein B mRNA editing catalytic polypeptide-like (APOBEC) mutational signatures [144,145]. These features likely contribute to neoantigen production and confer some sensitivity to ICI therapy. Otherwise, in cases of cHL and DLBCL that are driven by EBV infection, EBV-derived viral epitopes may provide foreign neoantigens that can drive T-cell responses [146].

### 3.6. Innate Immune Dysfunction

Aberrancies in the innate immune system also contribute to lymphoma cell survival. Beyond the MDSCs and TAMs discussed above, subversion of NK cell responses has also been demonstrated. Alterations in CD58 expression that impair NK cell recognition occur in approximately 60% of DLBCL cases, with the complete absence of surface CD58 in 21% [104]. In cHL, the HRS cells secrete soluble NK group 2D (NKG2D) ligand, which binds to NKG2D on circulating NK cells, where it induces internalization and downregulation of the receptor [147,148]. TGF-β secretion within the TME further reduces NKG2D expression and contributes to impaired NK cell immune surveillance [149,150]. Moreover, killer cell immunoglobulin-like receptor (KIR) is expressed on NK cells, where it interacts with HLA molecules to provide self-tolerance against NK-mediated cytotoxicity [151]. Thus, lymphoma cells with retained expression of HLA-I naturally shield themselves from NK recognition, making KIR an attractive target for blockade. Finally, neutrophils in the TME of cHL, DLBCL, and BL secrete a proliferation-inducing ligand (APRIL) that stimulates B-cell growth [152,153].

## 4. Engaging the Antitumor Immune Response

An increased understanding of immune evasion mechanisms has enabled the rational development of agents designed to re-engage the antitumor immune response. A host of immunomodulatory small molecules that target various aspects of B-lymphocyte development, survival, signaling, proliferation, and apoptosis has been prepared [11]. Since the advent of rituximab, therapeutic antibodies have become a mainstay of combination and maintenance therapy regimens [154,155,156]. Meanwhile, improvements in linker design and antibody engineering have prompted a resurgence of antibody–drug conjugates [157], while similar advancements in radiation therapy have facilitated the exploration of new radioimmunotherapies [158,159]. Vaccines to promote tumor antigen presentation on dendritic cells continue to be studied as well [160]. Finally, adoptive cell therapies have ushered in a new era of cellular immunotherapy, with chimeric antigen receptor (CAR) T-cells approved for several B-cell neoplasms and numerous additional CAR-T and CAR-NK cell products under clinical investigation [97,98,161].

The following sections of this review will focus on the clinical use of checkpoint inhibitors of both the adaptive and innate immune systems, immunostimulatory antibodies, and polyspecific engagers of T- and NK cells.

### 4.1. Immune Checkpoint Inhibitors

#### 4.1.1. Non-Hodgkin Lymphomas

Early studies of checkpoint inhibitors in advanced malignancies had underwhelming overall response rates (ORRs) [162,163]. However, the responses that were observed appeared durable, prompting further investigation. A phase I study of the anti-CTLA-4 antibody, ipilimumab, recruited 18 patients with relapsed or refractory (r/r) FL, DLBCL, and MCL (Table 1) [82]. While the ORR was again low (11%), one patient with DLBCL experienced a complete response (CR) lasting >31 months, while another patient with FL experienced a partial response (PR) lasting 19 months. A subsequent phase Ib trial (CHECKMATE-039) of the anti-PD-1 antibody, nivolumab, again showed a modest response in patients with r/r DLBCL and FL, with ORRs of 36% and 40%, respectively [90]. CRs were seen in 10% and 18% of patients with DLBCL and FL, respectively. Patients with other B-cell non-Hodgkin lymphomas (B-NHLs) did not respond. The median progression-free survival (PFS) in the B-NHL cohort was 23 weeks. Unfortunately, these findings could not be recapitulated in the larger phase II follow-up study, CHECKMATE-139 [83]. Patients with DLBCL, who either failed (*n* = 87) or were ineligible for autologous hematopoietic stem cell transplantation (auto-HSCT; *n* = 34), were treated with nivolumab, yielding ORRs of 10% and 3%, respectively. Only one patient with DLBCL and failed auto-HSCT experienced a CR (3%), and the median PFS was only 1.9 and 1.4 months, respectively. Likewise, the phase II follow-up study of nivolumab for r/r follicular lymphoma patients (*n* = 92) also showed minimal responses (ORR 4%) with short PFS (median 2.2 months) [164]. Similar results were obtained in a phase II trial of the anti-PD-1 antibody, pembrolizumab, in patients with r/r CLL or CLL with Richter transformation (RT) [165]. No objective response was seen in any patient with r/r CLL. However, CLL patients with RT had an ORR of 44% with one CR (11%). The median PFS was also longer in the RT group at 5.4 months compared to 2.4 months in the r/r CLL subset. Ultimately, these studies demonstrated that ICI monotherapy was inadequate in the majority of B-NHLs.

More encouraging results came in a small phase I study of nivolumab in patients with PCNSL and PTL, where a 100% ORR was achieved, including CRs in three of four PCNSL patients and the sole PTL patient [72]. The KEYNOTE-013 trial demonstrated the efficacy of pembrolizumab in 21 patients with r/r PMBCL, with an ORR of 48% that included CRs in 33% [70,71]. These responses were reasonably durable, with a median PFS of 10.4 months. The KEYNOTE-170 trial validated these results, again demonstrating an ORR of 45% amongst 53 patients with r/r PMBCL treated with pembrolizumab [70]. However, the CR rate (CRR) was slightly lower at 13%, and the median PFS was reduced to 5.5 months. Still, the median duration of response was not reached after 29.1 months of follow-up in KEYNOTE-013 and 12.5 months of follow-up in KEYNOTE-170. Moreover, no patient with a CR in either study experienced progression, including two patients who continued to be followed for at least one year off therapy. Collectively, these results indicate that ICI can be effective in patients with certain DLBCL subtypes.

Following the disappointing results of ICI monotherapy in indolent B-NHL, several studies have evaluated ICI therapy as part of combination regimens. Prompted by the dramatic results seen with dual checkpoint inhibitor therapy in solid tumors, one cohort of the CHECKMATE-039 trial evaluated the combination of nivoumab (PD-1) and ipilimumab (CTLA-4) in 15 patients with r/r B-NHL [5]. No CRs were achieved, and the ORR was merely 20%, with a median PFS of 1.5 months. Unfortunately, these results were not significantly improved compared to ICI monotherapy. In contrast, several studies have demonstrated benefit when ICI is combined with anti-CD20 antibodies. A phase II study of pidilizumab (purportedly anti-PD-1) plus rituximab achieved an ORR of 66% in patients with r/r FL [166]. CRs were seen in 15 of 29 patients (52%). These response rates appeared to be slightly improved compared to historical results with rituximab alone, which encouraged subsequent combination therapy trials. Accordingly, the combination of atezolizumab (anti-PD-L1) plus obinutuzumab provided an ORR of 57% in patients with r/r FL, although the ORR was lower in those with r/r DLBCL (16%) [167]. Meanwhile, the combination of pembrolizumab and rituximab in r/r FL was especially encouraging, with a CRR and ORR of 60% and 80%, respectively, on interim analysis [168]. Conversely, combining ipilimumab with rituximab was less effective, demonstrating ORRs of 38% in r/r FL and only 3% in other B-NHLs [169]. Median PFS was only 2.6 months in this study. This observation appears consistent with the results from the monotherapy trials, suggesting that PD-1/PD-L1 inhibition may be more effective than CTLA-4 blockade in combination with CD20-directed therapy.

Building on these results, the incorporation of additional agents into the combination regimen has been shown to provide even further benefit. For example, the addition of bendamustine to atezolizumab plus obinutuzumab was assessed in a phase Ib/II trial in 15 patients with previously untreated FL [170]. CRs were seen in 67%, with an ORR of 80%, with both metrics improved when compared to the results obtained in the absence of bendamustine [167]. Expanding on this approach, the addition of atezolizumab to R-CHOP (rituximab, cyclophosphamide, doxorubicin, vincristine, and prednisone) in patients with untreated DLBCL afforded an ORR of 88%, with 78% of patients achieving CR [171]. This compares favorably to R-CHOP alone, and the adverse events were manageable (most commonly neutropenia). Recent results of pembrolizumab plus R-CHOP were also encouraging, with an ORR of 90% and CRs in 77% of patients with untreated DLBCL and high-grade FL [172]. PFS was 83% after 24 months of follow-up.

Modest results have also been seen when combining ICI therapy with Bruton’s tyrosine kinase inhibitor, ibrutinib. A combined phase I/II study of nivolumab plus ibrutinib in patients with CLL/SLL, FL, and DLBCL garnered response rates that were comparable to ibrutinib alone [173]. However, patients with CLL that had undergone RT did relatively well, with a CR achieved in 10% of patients and an ORR of 65%. The median PFS in this group was 5.0 months. Results obtained from the combination of durvalumab (anti-PD-L1) plus ibrutinib in r/r FL and DLBCL were similar, with ORRs of 13–35% across the subgroups [174]. Otherwise, combinations of ICI with the immunomodulator lenalidomide have met with dose-limiting toxicities (DLTs) [175].

Finally, the encouraging results of ICI therapy in PMBCL were further bolstered by the addition of the antibody–drug conjugate (ADC) brentuximab vedotin (BV). As part of the combined phase I/II trial CHECKMATE-436, nivolumab plus BV was administered to 30 patients with r/r PMBCL [176]. This regimen provided an ORR of 73%, with CR documented in 37%. The PFS was 63.5% at the six-month follow-up, although 53% of patients had expected grade 3–4 toxicities (predominantly cytopenias). There were no treatment-related deaths.

#### 4.1.2. Hodgkin Lymphoma

In stark contrast to NHL, ICI monotherapy has been highly effective in cHL (Table 2). The initial phase I study of nivolumab demonstrated an 87% ORR amongst 23 patients with r/r cHL [66]. CR was achieved in 17%, and these responses appeared durable, with a PFS of 86% at 24 weeks. The follow-up phase II trial, CHECKMATE-205, confirmed the high response rates, with a CRR and ORR of 16% and 69%, respectively [67,173]. The median PFS was 14.7 months in this cohort of 243 patients with r/r disease. Similar results were seen in a small (*n* = 16) trial of Japanese patients with r/r cHL, where the CR and ORRs were 31% and 88%, respectively, with a median PFS of 11.7 months [177,178]. CHECKMATE-205 was later expanded to include 51 patients with previously untreated stage IIB or higher cHL [179]. In this front-line setting, nivolumab monotherapy afforded an ORR of 69%, with 18% of patients achieving CR. Patients then continued on to receive additional nivolumab in combination with doxorubicin, vinblastine, and dacarbazine (AVD), which further improved the ORR and CRR to 84% and 80% at the end of treatment, respectively. Moreover, the PFS was 92% at the nine-month follow-up.

Beyond nivolumab, several other ICIs have been evaluated as monotherapy for cHL, with largely similar results. The KEYNOTE-013 (phase Ib) [180] and KEYNOTE-087 (phase II) [181,182] trials examined the use of pembrolizumab in adults with r/r disease. The ORR in these trials was 65–72%, with CRs seen in 16–28%. At the end of the planned follow-up period, the median PFS was 13.7 months. Tislelizumab (anti-PD-1) monotherapy was also shown to be effective in a phase II trial of 70 patients with r/r cHL; the ORR was 87%, with an impressive CRR of 63% [183]. The PFS was 75% at nine months. Sintilimab (anti-PD-1) was also evaluated in a phase II trial (ORIENT-1) for r/r cHL, where an ORR of 80% was seen [184]. Finally, in a phase II trial of the anti-PD-1 antibody camrelizumab with 75 patients with r/r cHL, the ORR was 72%, with CR documented in 28% [185]. Median PFS was 11.3 months per investigator assessment (PFS was not reached when assessed by the independent review committee).

Peculiarly, response rates to ICI monotherapy are more variable in children and young adults. For instance, a phase I/II trial (ADVL1412) of single-agent nivolumab produced much lower response rates [186]. Amongst the 10 evaluable patients with cHL, the ORR was merely 30%, and only one (10%) achieved a CR. Results of atezolizumab monotherapy were also underwhelming in a small cohort of cHL patients in the iMATRIX trial, where the ORR was only 22%, and no patients achieved a CR [187]. In contrast to nivolumab and atezolizumab, however, the efficacy of pembrolizumab did largely translate into the pediatric population, with a phase I/II study (KEYNOTE-051) in children with r/r cHL yielding an ORR of 60% and median PFS of 12.2 months [188]. More work is needed to elucidate the mechanisms that account for these differences amongst age groups and agents.

Similarly inspired by responses seen in advanced solid malignancies, the combination of nivolumab and ipilimumab was studied in a phase Ib trial of 31 patients with r/r cHL (CHECKMATE-039) [5]. The results were similar to those seen in nivolumab therapy alone, although toxicity was somewhat increased. Thus, subsequent studies have focused on combining ICI therapy with the antibody–drug conjugate brentuximab vedotin (BV). A phase I/II trial of nivolumab plus BV in r/r cHL showed impressive responses, with 61% of patients achieving a CR for an ORR of 82% [189]. As with ICI monotherapy, the responses were durable, with 89% PFS at 6 months. Infusion reactions were common with BV (44% of patients), but overall adverse events were mild and predominantly grades 1 and 2. An ACCRU trial (phase II) then assessed the combination of nivolumab and BV as front-line therapy in older (age > 60 years) patients and younger (age <60 years) patients ineligible for standard ABVD (doxorubicin, bleomycin, vinblastine, and dacarbazine) chemotherapy [190]. While the trial failed to reach predefined activity criteria by the time of interim analysis, the evaluation of the 46 enrolled patients demonstrated an ORR of 61%, with CR seen in 22%. Again, the responses were highly durable, with a median PFS of 18.3 months. Treatment was well tolerated in this population, and thus despite failure to meet the trial activity criteria, nivolumab plus BV may be an attractive option for patients unable to tolerate conventional chemotherapy.

The NIVAHL trial then sought to combine nivolumab with AVD chemotherapy or to sequence the nivolumab shortly before AVD in patients with newly diagnosed, early-stage (stage I–II), unfavorable risk cHL [191]. Both approaches resulted in high remission rates, with concomitant therapy offering an ORR of 100%, with an 83% CRR. Sequential therapy was also efficacious, with an OR documented in 98% and CR in 84%. Moreover, on interim analysis, high rates of remission (CR 51%, ORR 96%) were seen after the four doses of nivolumab monotherapy that preceded systemic AVD. PFS at 12 months was outstanding in both the concomitant and sequential therapy groups, at 100% and 98%, respectively.

Finally, a recent phase I/II trial assessed the combination of BV with either nivolumab, ipilimumab, or both (triplet therapy) in the r/r disease setting [192]. ORRs were similar between the three groups at 89%, 76%, and 82%, respectively. Likewise, CRRs were not significantly different at 61%, 57%, and 73%, respectively, although there was a trend toward higher efficacy in the triplet therapy group. The rates of adverse events were slightly higher in the nivolumab and triplet therapy cohorts, although these were also interpreted as the most active regimens in the study. Thus, these two interventions are being further compared in an ongoing follow-up study (NCT01896999).

#### 4.1.3. T-Cell Lymphomas

As in B-cell lymphomas, checkpoint inhibition in T-cell malignancies has been met with mixed responses (Table 3). In addition to PD-1, malignant T-cell clones can also gain expression of PD-L1 [193], with different frequencies of expression across the T-cell lymphoma subtypes [194]. For example, PD-L1 expression is commonly (>50% of cases) seen in peripheral T-cell lymphomas (PTCL) such as NK/T-cell lymphoma (NKTCL), angioimmunoblastic T-cell lymphoma (AITL), and anaplastic lymphoma kinase (ALK)-negative anaplastic large cell lymphoma (ALCL) [194,195]. In NKTCL, the high expression frequency is closely associated with EBV infection [196,197]. In contrast, PD-1 expression in cutaneous T-cell lymphomas (CTCLs) varies with the stage of disease [198].

As in B-cell lymphomas, PD-L1 expression in the TME may correlate with clinical responses to ICI therapy, although the number of treated patients with T-cell lymphomas remains small. In a small retrospective analysis of seven patients with NKTCL treated with pembrolizumab, the ORR was 100%, with 71% of patients achieving CR [199]. A similar retrospective analysis of another seven NKTCL patients receiving pembrolizumab was reported the following year, with slightly lower CR and OR rates of 29% and 57%, respectively [200]. Encouraging results have also been reported with pembrolizumab in follicular T-cell lymphoma (FTL) [201] and nivolumab in ALK-negative ALCL [202].

Meanwhile, responses of cutaneous T-cell lymphomas (CTCL), including mycosis fungoides (MF) and Sezary syndrome (SS), have been more modest. Early stages of the CHECKMATE-039 trial enrolled patients with both PTCLs and CTCLs. When treated with single-agent nivolumab, ORs were seen in 40% of patients with PTCL and 15% with MF; no CRs were achieved [90]. When the combination of nivolumab and ipilimumab was used, only one partial response was seen amongst 11 patients (ORR 9%) with unspecified T-cell lymphomas [5]. Subsequent trials of pembrolizumab in MF have yielded slightly improved ORRs of 33–56% [201,203].

The precise role of the PD-1/PD-L1 axis in T-cell lymphoma remains unclear and is complicated by dual-expression of the malignant T-cells. Clinically, this has manifested as a paradoxical hyperprogression of disease in patients with adult T-cell leukemia/lymphoma (ATLL) treated with a PD-1 inhibitor [204]. In this phase II trial of nivolumab for ATLL with evidence of PD-L1 overexpression, the first three patients treated experienced rapid disease progression, leading to termination of the study. While the precise mechanism by which PD-1 inhibition drove disease progression remains unclear, multiple explanations have been proposed and are under investigation [205].

#### 4.1.4. Peritransplant Setting

Checkpoint inhibition has been studied in the setting of HSCT in an attempt to bolster the graft-versus-malignancy (GVM) response of the transplanted T-lymphocytes (Table 4). The CTEP 6082 trial (phase I) enrolled 17 patients with either cHL (*n* = 14) or B-NHL (*n* = 3) who had relapsed at least 90 days after an allogeneic HSCT (allo-HSCT) [206]. Escalating doses of ipilimumab were administered as a single infusion, and no DLTs were observed even at the highest planned dose (3.0 mg/kg). Specifically, there was no evidence of grade 3 or 4 graft versus host disease (GVHD) in any patient. Unfortunately, the response rates in this dose-finding study were also underwhelming, with an ORR of only 14% and 33% in cHL and B-NHL patients, respectively. Given the apparent safety, however, a follow-up phase I/Ib trial assessed the administration of multiple doses of ipilimumab at either 3 or 10 mg/kg in patients who relapsed more than 90 days after allo-HSCT [207]. The trial included a small number of both cHL (*n* = 7) and B-NHL (*n* = 4) patients, but ORRs in these patients were again poor at 14% and 0%, respectively. Notably, no patients who received the lower dose of ipilimumab had an objective response despite repeated dosing. The incidence of GVHD and immune-related adverse events was higher in the 10 mg/kg cohort.

Expanding on these results, a phase II trial evaluated the combination of ipilimumab plus lenalidomide in patients with lymphoid malignancies that relapsed after allo-HSCT and in high-risk patients who had undergone auto-HSCT within the past six months [208]. Included in these cohorts were eight and six B-NHL patients, respectively. While the ORRs were similar amongst the two groups (75% and 83%), auto-HSCT was associated with higher CRRs at 83% vs. 38%. Likewise, the 12-month PFS was also higher in the auto-HSCT cohort at 86% compared to 56% in the allo-HSCT cohort. Only one patient in the allo-HSCT group experienced GVHD, which was a flare of a preceding episode. A total of four patients experienced grade 4 neutropenia that required a dose-reduction of lenalidomide. Otherwise, there were no significant differences in adverse events between the two groups.

PD-1 blockade has been assessed as an early adjunct to auto-HSCT to reduce immune tolerance. In a phase II trial of patients with DLBCL undergoing auto-HSCT, pidilizumab was administered every 42 days for three cycles, beginning 30–90 days from transplant [209]. The PFS at 16 months was 72%, which compared favorably to the 18-month post-allo-HSCT PFS of 52% in a historical cohort of 46 patients at the trial authors’ institutions who would have otherwise met the eligibility criteria. In a subsequent phase II trial, pembrolizumab was given every three weeks for eight cycles, beginning within 60 days of auto-HSCT for DLBCL [210]. The overall PFS at 18.5 months was 58%, which did not meet the prespecified efficacy criteria. Indeed, this value reflects the PFS of the aforementioned historical comparison cohort and, as discussed above, further suggests that PD-1 blockade is not effective in unselected DLBCL. That the post-auto-HSCT trial with pidilizumab did meet the prespecified efficacy criteria could reflect that pidilizumab may, in fact, target delta-like protein 1 and not PD-1, as previously thought [211].

#### 4.1.5. Checkpoint Inhibitors under Investigation

Beyond the traditional checkpoint molecules, several other receptors are under investigation as therapeutic targets in hematologic malignancies—including TIM-3, LAG-3, and TIGIT. Early studies showed that IL-12 upregulates TIM-3 and LAG-3 expression on intratumoral T-cells in patients with follicular lymphoma, serving as markers of T-cell exhaustion and functional impairment [139,212]. Moreover, TIM-3 and LAG-3 are nearly always expressed in the TME of cHL (>96% of cases) [213]. In both DLBCL and FL, expression of these ligands on TILs has been correlated with a decrease in treatment efficacy and overall survival [34,118,212,214]. Preclinical studies have shown enhanced cytotoxic and antitumor T-cell responses in the presence of anti-TIM-3 and anti-LAG-3 antibodies [34,215]. Similarly, TIGIT is abundant on intratumoral Tregs and follicular dendritic cells in FL [126,216]. Variable expression is also seen on exhausted T-effector cells in both cHL and B-NHL [217,218]. As with TIM-3 and LAG-3, increased prevalence of TIGIT+ T-cells is associated with reduced survival in B-cell lymphomas [216]. Accordingly, clinical trials assessing inhibitors of all three targets in lymphoma are underway (Table 5).

### 4.2. Checkpoint Inhibitors of the Innate Immune System

As with T-cells, there has been an intense effort to modulate macrophage and NK cell function so as to elicit an antitumor innate immune response (Table 6). One regulatory axis of therapeutic interest is the interaction between macrophage SIRPα and its inhibitory ligand CD47. As previously discussed, lymphoma cells overexpress CD47, thereby suppressing TAM-mediated phagocytosis and contributing to macrophage dysfunction within the TME. In patient-derived murine xenograft models of acute myeloid leukemia (AML), an anti-CD47 antibody completely eradicated AML and provided long-term disease-free survival [219]. Likewise, the combination of anti-CD47 and anti-CD20 antibody therapy led to the eradication of NHL in engrafted mice [219].

Based on these results, the combination of the anti-CD47 antibody Hu5F9-G4 and rituximab was tested in a phase Ib trial of B-NHL [137]. A total of 22 patients with either DLBCL (*n* = 15) or FL (*n* = 7) were enrolled, and the ORR was 50%, with 36% achieving a CR. Patients with FL had higher response rates than those with DLBCL, although the PFS was remarkably high in both groups. No clinically significant adverse events were observed. Thus, Hu5F9-G4 (magrolimab) has proceeded for further evaluation in a phase II trial (NCT02953509).

TTI-621 is a decoy receptor composed of the CD47-binding domain of human SIRPα fused to the Fc region of human IgG1 [220]. TTI-621 serves to neutralize inhibitory CD47 signaling while simultaneously activating macrophages via the Fc receptor [221]. Early trials showed a high response rate in patients with Sezary syndrome (cutaneous T-cell lymphoma) [222]. A large phase I trial recently assessed TTI-621 as monotherapy and in combination with either rituximab or nivolumab in patients with relapsed hematologic malignancies [223]. The ORR for all 164 patients enrolled in the trial was 13%. Amongst patients who received TTI-621 monotherapy, those with DLBCL had the highest response rate (2/7, 29%). A small number of patients with cHL received TTI-621 plus nivolumab (*n* = 4), and one patient each achieved complete and partial responses for an ORR of 50% in this small cohort. However, this is not significantly different from what would be expected for nivolumab monotherapy in r/r cHL. Treatment-related adverse events (AEs) occurred in 80% of patients, with serious AEs in 17% (predominantly cytopenias).

A similar molecule composed of the CD47 binding domain fused to the Fc region of human IgG4, TTI-622, has recently been reported with some preliminary efficacy early in the phase I dose-escalation trial [224]. Likewise, CC-90002 is another anti-CD47-IgG4 fusion protein that has been evaluated in combination with rituximab. Early results from a phase I study of 24 patients with r/r cHL showed only modest results, with one CR (4%) and an ORR of 13% [225]. Notably, both TTI-622 and CC-90002 were specifically engineered to reduce binding to red blood cells and resultant hemolytic anemia. While cytopenias were common, no instances of hemolysis were reported in either study.

The interaction between KIR on NK cells and HLA molecules on lymphoma cells is also being explored as a therapeutic target, as the KIR–HLA interaction provides self-tolerance against NK-cell-mediated cytotoxicity. The importance of KIR is underscored by the role of KIR–MHC mismatch in allo-HSCT, which serves to enhance the graft-versus-leukemia effect. An early phase I trial of the anti-KIR antibody lirilumab included 17 patients with CLL or NHL [226]. The safety profile was acceptable, with no DLTs seen and no maximum tolerated dose (MTD) reached. However, no objective response was seen in any of the patients enrolled. A follow-up phase Ib study combined lirilumab with nivolumab in patients with cHL, B-NHL, and T-NHL [227]. Unfortunately, the results were similarly underwhelming, without increased objective improvement when compared to nivolumab monotherapy in these diseases.

### 4.3. Immune Checkpoint Stimulators

**Figure 3:**
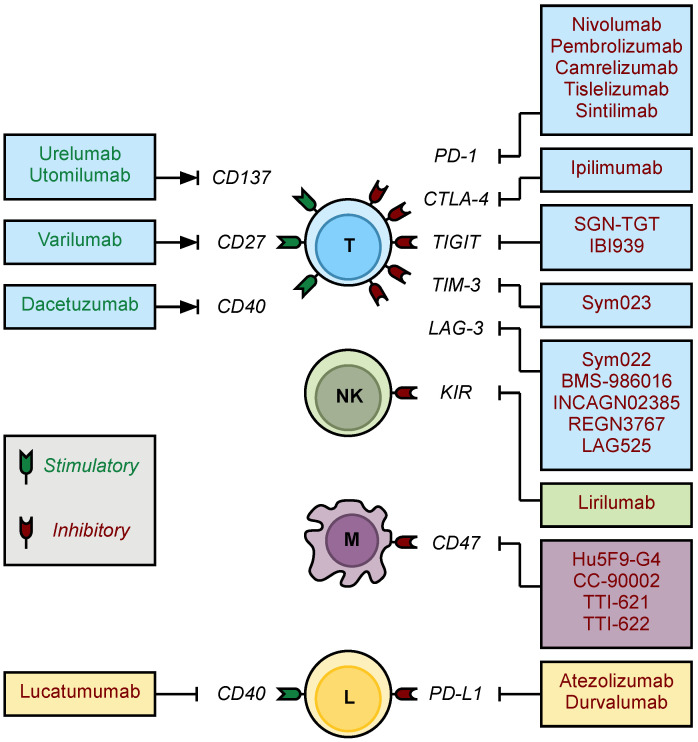
Immune checkpoint inhibitors and stimulators in lymphoma. Numerous checkpoint inhibitors and stimulators are in clinical use or under investigation in lymphomas. Putative targets on T-cells (T), natural killer cells (NK), tumor-associated macrophages (M), and lymphoma cells (L) have been identified. Green shading and text denote stimulatory receptors and antibodies, respectively. Likewise, red shading and text denote checkpoint inhibitor proteins and antibodies, respectively.

Rather than block checkpoint inhibition, a complementary strategy is to promote immune activation using stimulatory antibodies. Several costimulatory ligands on T-cells have been identified, including CD137 (4-1BB), CD27, and CD40 (Figure 3). Preclinical studies have shown that engaging these ligands with agonistic antibodies leads to increased T-cell proliferation, inflammatory cytokine release, and myeloid and NK cell recruitment, all of which contributed to robust antitumor immune responses [228,229,230,231]. Thus, immunostimulatory antibodies that engage these molecules have progressed to early-phase clinical trials (Table 7).

CD40 was one of the earliest costimulatory targets to be assessed in lymphoma when a phase I trial of dacetuzumab (SGN-40) monotherapy enrolled 50 patients with r/r disease [232]. While dacetuzumab was well-tolerated, only six objective responses were seen (ORR 12%). Likewise, a small trial of dacetuzumab monotherapy in CLL was also disappointing, producing no responses [233]. In the follow-up phase II study, which primarily enrolled patients with r/r DLBCL, response rates were modest at best (9%), and the median PFS was 36 days [234]. Subsequent trials investigated the use of dacetuzumab in combination with rituximab and chemotherapy; however, the ORRs seen in these trials were comparable to those of the parent regimens alone, and, thus, no benefit from concurrent CD40 stimulation was readily apparent [235,236].

Given that CD40 is highly expressed on the cell surface of many B-cell malignancies, conventional antibody targeting to drive antibody-dependent cellular cytotoxicity (ADCC) has also been explored. Lucatumumab is a fully human, antagonistic anti-CD40 antibody developed for this purpose. A phase I trial of lucatumumab monotherapy in 24 patients with CLL produced an ORR of 4% and identified an MTD of 3.0 mg/kg, with asymptomatic elevations in amylase and lipase seen at higher doses [237]. The follow-up study expanded enrollment to include patients with DLBCL, indolent B-NHLs, and cHL [238]. Reasonable ORRs were seen in patients with FL and MALT lymphoma, at 33% and 43%, respectively, suggesting that CD40 targeting may be beneficial in patients refractory to rituximab-based regimens.

Urelumab and utomilumab are two agonistic antibodies that activate CD137 (4-1BB). Early studies suggested that monotherapy with each of these agents was well tolerated, although transaminitis was seen with higher doses of urelumab. Outcomes data for the patients with lymphoma were not reported [239,240]. Two follow-up trials assessed the combination of each agent alongside rituximab in patients with B-NHL. Unfortunately, the ORRs for the urelumab and utomilumab combination regimens were modest (20% and 21%, respectively), which were not significantly improved compared to historical results with rituximab alone or other standard of care regimens [241,242].

Finally, results of a phase I trial of the CD27 agonist varlilumab (CDX-1127) were recently reported [243]. CD27 is expressed on unstimulated T-cells, B-lymphocytes, and nearly all subtypes of mature B-cell lymphomas [244,245]. Therefore, varlilumab is designed to invoke an antitumor response via enhanced T-cell activation as well as ADCC [246,247], and potent antilymphoma activity was seen both in vitro and in vivo [248,249]. The phase I trial enrolled 34 patients with r/r B-cell and T-cell malignancies, and patients were treated with escalating doses of varlilumab. No DLTs were observed, and only one grade 3 treatment-related AE was reported (transient elevation in serum alkaline phosphatase). The only documented response to treatment was a CR in one patient with stage IV cHL (ORR 3%), who had previously failed induction therapy and progressed through four subsequent lines of treatment (including HSCT). The CR was sustained at the final follow-up appointment >33 months after study enrollment. An additional five patients experienced stable disease. Thus, while varlilumab is unlikely to provide significant benefit as a single agent, future studies combining varlilumab with either nivolumab (for cHL) or rituximab (for B-NHL) are already underway (NCT03038672).

### 4.4. Polyspecific Engagers

**Figure 4:**
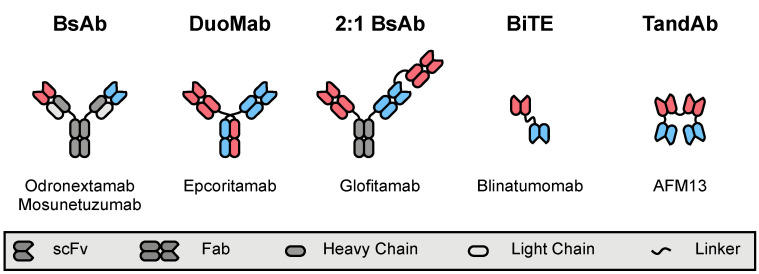
Multivalent antibody constructs for lymphoma immunotherapy. Numerous polyspecific antibody constructs with a range of binding valencies have been developed, and several formats are in advanced stages of development for lymphoma. Bispecific antibodies (BsAbs) are a very common format, with multiple derivatives, including DuoMabs and multivalent ligands incorporating additional antigen-binding fragments (Fabs). Bispecific T-cell engagers (BiTEs) and tandem diabodies (TandAbs) are physically smaller targeting ligands based on the careful fusion and folding of single-chain variable fragments (scFvs). Here, red and blue elements distinguish scaffold components that correlate with binding to two distinct target epitopes.

Another strategy to promote an antitumor immune response is to redirect effector cells to the malignant target cells. This can be accomplished by using a ligand—typically antibody-derived—that binds to cell surface proteins on both cell types. Typically, the substrate on the effector cell is a stimulatory receptor (such as CD3 on T-cells), whereas the target is a tumor-associated antigen. As the primary cytotoxic effector cells of the adaptive and innate immune systems, numerous formats of polyspecific engagers have been developed to redirect T-cells and NK cells, respectively [250]. The following sections will review the clinical use of various bispecific antibody (BsAb) formats in lymphoma (Figure 4).

#### 4.4.1. T-Cell-Engaging Formats

A multitude of antibody formats with different antigen valencies have been developed and evaluated in clinical trials (Table 8) [251]. While antibodies with divergent specificities have been investigated for decades, contemporary advances in molecular biology, protein engineering, and antibody production have fostered an exponential growth of BsAb development. In particular, these techniques fostered the construction of the BiTE blinatumomab [252]. Blinatumomab is comprised of two single-chain variable fragments (scFvs)—one binding to CD3 and the other to CD19—recombinantly fused by a peptide linker, and it has shown considerable activity in B-cell acute lymphoblastic leukemia, for which it was FDA-approved in late 2014 [253].

The final results of the principal phase I trial of blinatumomab in lymphoma were reported in 2016 and showed considerable efficacy in patients with r/r FL (ORR 80%), MCL (ORR 71%), and unselected DLBCL (ORR 55%) [254]. Similar results were obtained in a phase II study for r/r DLBCL, where 19% of patients achieved CR for an ORR of 43% [255]. The median PFS was 3.7 months. Another phase II study assessed blinatumomab as salvage therapy in multiple B-NHL subtypes, albeit most with DLBCL (*n* = 34 of 41 total patients), and showed an ORR of 37% [256]. Complete responses were seen in 22% of patients, and the median PFS in this subgroup was 8.4 months.

While results with single-agent blinatumomab were encouraging, most results were partial and of limited duration. Improved response rates were seen using the combination of blinatumomab and lenalidomide. In a phase I trial of 18 patients with B-NHL (including DLBCL, FL, MCL, and MZL), this combination produced an ORR of 83% and CRs in 50% [257]. The median PFS was analogous to earlier trials at 8.3 months. Blinatumomab has also been studied as consolidation following rituximab-based chemoimmunotherapy (e.g., R-CHOP). In a phase II study, 28 patients with high-risk DLBCL received six cycles of rituximab chemotherapy during a run-in period, followed by escalating doses of blinatumomab [258]. The ORR was 89% in this high-risk group, and 93% of patients who completed the trial therapy were still alive after the median follow-up of 8.6 months. An ongoing phase II study is also examining the use of blinatumomab, following debulking R-CHOP therapy in patients with CLL that has undergone Richter transformation. Preliminary results from five patients have indicated CRs in two patients (50%) and a PR in a third patient (ORR 60%) [259].

One limitation to BiTE therapy is that the small size of the targeting agent leads to rapid clearance, and, thus, blinatumomab needs to be administered via continuous intravenous infusion. Larger antibody-based formats do not have this limitation and, therefore, remain an attractive alternative. Odronextamab (REGN1979) is a full-length, hinge-stabilized IgG4 construct with bispecificity for CD3 and CD20. Early results from an ongoing phase I trial showed considerable efficacy in patients with r/r FL (*n* = 7), with an ORR of 100% and a CR documented in 71% [260]. Results in patients with r/r DLBCL were initially modest but improved considerably as the dose of odronextamab was escalated, including attainment of CR in patients refractory to CAR T-cell therapy [261]. As the trial has progressed, ORRs in patients with FL remained high (ORR >93%) with a median PFS of 12.8 months [262]. At higher doses, CRs have been seen in 60% of patients with DLBCL who have not had prior CAR T-cell therapy and in 24% of those refractory to CAR T-cells (ORR 60% and 33%, respectively) [262]. Based on these results, an international phase II trial of odronextamab in r/r DLBCL is underway.

Mosunetuzumab (RG7828) is another full-length antibody (IgG1) with bispecificity for CD3 and CD20. Interim results of a large, ongoing, phase I trial in patients with heavily pretreated (including CAR T-cell therapy), aggressive (*n* = 119), and indolent B-NHLs (*n* = 64) have been reported, with ORRs of 35% and 64%, respectively [263]. Considerable efficacy was seen in patients with multiple-relapsed FL (*n* = 62), where 50% of patients achieved a CR for an ORR of 68% and median PFS of 11.8 months [264]. Mosunetuzumab has also been assessed as first-line therapy in patients with DLBCL who are not candidates for standard of care chemoimmunotherapy. Treatment was well tolerated and offered an ORR of 58% and a CRR of 42% in this population [265]. Finally, mosunetuzumab has been combined with CHOP chemotherapy (M-CHOP) in patients with both r/r B-NHL and untreated DLBCL. High ORRs (86% and 96%, respectively) and CRRs (71% and 85%) were seen in these groups, though the specific contribution of mosunetuzumab to the regimen and how it compares to R-CHOP remain to be clarified.

Glofitamab (previously RG6026 and CD20-TCB) is a BsAb construct with a unique format that incorporates one additional Fab fragment [266]. This raises the binding valency of the BsAb to three, with two paratopes targeting CD20 and the third recognizing CD3. The increased avidity prolongs the half-life and increases the potency of glofitamab both in vitro and in vivo [266]. An ongoing phase I/Ib trial is evaluating the combination of glofitamab and obinutuzumab in patients with r/r B-NHL. Interim results of the dose-finding series demonstrated an ORR of 90% with CRs in 80% of patients treated with the highest tested dose [267]. High response rates were again seen in a follow-up series, although patients with indolent B-NHL (CRR 75%, ORR 100%) responded better than those with aggressive B-NHL (CRR 29%, ORR 50%).

Cytokine release syndrome (CRS) is a significant side effect of treatment with T-cell-directing antibody constructs. Subcutaneous (SQ) administration has been proposed as a method of altering the pharmacokinetics in a manner that minimizes the incidence of CRS. Clinical data regarding SQ dosing is available for two constructs—epcoritamab and mosunetuzumab. Epcoritamab (GEN3013) is a CD3 × CD20 BsAb that was designed for SQ administration [268]. A phase I/II trial enrolled 67 patients with heavily pretreated DLBCL, FL, or MCL [269]. While CRS was reported in 58% of patients, all instances were grade 1–2 in severity, and no patient discontinued treatment as a result. Efficacy was also maintained, with ORR of 71% amongst the 28 evaluable patients. A phase I/Ib trial of mosunetuzumab SQ reported similar results, with grade 1–2 CRS events seen in 35% of patients [270]. Compared to mosunetuzumab IV, where 15% of patients experienced grade 2 CRS at doses of 0.05–2.8 mg [263], no grade 2 CRS occurred in the SQ cohort at doses <13.5 mg. Moreover, the ability to safely administer higher doses may have contributed to the higher ORR seen with SQ dosing compared to IV (68% vs. 45%), although the number of patients in the SQ trial was considerably smaller. Higher ORRs were seen in patients with indolent B-NHL, regardless of administration route.

Collectively, these trials have demonstrated that T-cell-engaging antibody constructs can elicit potent antitumor immune responses, even in highly refractory disease. Efficacy appears to be highest in patients with indolent lymphoma compared to aggressive subtypes, though many of these trials are still ongoing. Moreover, data regarding duration of response and PFS are limited and will require additional follow-up.

#### 4.4.2. NK-Cell-Engaging Formats

NK cell redirection has also been pursued in lymphoma (Table 9). Attempts to target innate effector cells to CD30+ HRS cells began decades ago, but the development of these early polyspecific ligands was halted due to production challenges [271,272,273]. Renewed interest in NK-cell-directing therapies has paralleled advances in antibody manufacturing, leading to the development of AFM13. AFM13 is a tetravalent, chimeric antibody construct based on tandem diabodies (TandAbs), with bispecificity for CD16A and CD30 [274]. The first-in-human phase I study of AFM13 enrolled 26 patients with r/r cHL who were subsequently treated with escalating doses of TandAbs. Adverse events were mild, and the MTD was not reached. However, of 26 evaluable patients, only three demonstrated an objective response (ORR 12%), with no CRs. A follow-up phase II trial was attempted but was terminated early due to low recruitment. At the time of trial closure, 24 patients with r/r cHL had been treated with AFM13, with one documented CR and three additional PRs (ORR 17%). The estimated PFS was 12.6% at 12 months.

A subsequent phase Ib study combined AFM13 with pembrolizumab in 30 patients with r/r cHL [275]. Treatment was well-tolerated, and an ORR of 83% was observed, with CR achieved in 37%. PFS reported at interim analysis was 77% at 6 months [276]. While these results suggest that there is benefit from the addition of AFM13 to pembrolizumab (compared to pembrolizumab monotherapy [182]), this small trial was not designed to delineate this difference. Additional trials of AFM13 are ongoing, and novel bispecific and trispecific killer engager constructs (BiKEs and TriKEs, respectively) are under development for a variety of hematologic malignancies [277,278,279].

## 5. Remaining Hurdles and Future Directions

While the characterization of inflamed and noninflamed lymphoma subtypes has helped stratify those diseases that are more likely to respond to checkpoint inhibitor therapy, there remains a need to identify accessible biomarkers predictive of outcome. Splice variations and amplifications of chromosome *9p24.1* have been identified in cHL and select inflamed DLBCL subtypes, contributing to PD-L1 overexpression and subsequent sensitivity to ICI therapy in these cases [12,73,74,75,110]. However, this correlation is inconsistent in many lymphoma subtypes, and considerable responses have been documented even in patients with low PD-L1 expression [173,280]. Moreover, the inflammatory cell infiltrate in cHL is skewed towards exhausted Th1 cells that lack PD-1 expression, while immunosuppressive Tregs comprise the predominant PD-1+ population [63,64]. Thus, the role of environmental T-cells in inflamed lymphomas is likely distinct from those in solid malignancies, further complicating the identification and interpretation of cytotoxic TILs. As such, many early phase trials are incorporating additional investigative arms and subanalyses aimed at identifying biomarkers that are more sensitive and specific for those patients who will benefit from ICI therapy.

MSI and overall TMB have been shown to be important drivers of neoantigen production and ICI response in solid tumors [281,282,283]. Unfortunately, TMB is inherently low in most lymphomas, with the exception of cHL and PMBCL [143,284]. Putative mechanisms that contribute to increased mutational load in these settings include viral infection (particularly EBV) and altered nucleic acid processing. A potential option to boost neoantigen production may be to combine ICI therapy with idiotype vaccines.

An attractive strategy to further improve polyspecific targeting constructs is through affinity and avidity optimization. Fcγ receptor engineering is a commonly employed strategy to modulate the ADCC activity and pharmacokinetics of antibodies and drug conjugates [285,286]. More recently, advances in molecular biology have enabled the production of polyvalent antibody constructs such as 2:1 BsAbs and TandAbs, among others [251,287]. However, the valency of antibody-based constructs has largely been restricted in the range of 2–4 binding paratopes. In vitro and in vivo studies of nonantibody-binding platforms with expanded valencies have shown improved tumor selectivity and binding efficacy when higher numbers of reduced affinity ligands are used [288,289,290]. Similar improvements in selectivity and safety have been seen in CAR T-cell studies, where high CAR expression is balanced with reduced scFv affinity [291,292,293,294,295]. Thus, as recombinant biology and protein manufacturing continue to improve, the development of polyvalent targeting scaffolds may become more attractive.

There has also been high interest in combining immunotherapy with small molecule modulators of immune cells. Lenalidomide is an immunomodulator that binds to the E3 ubiquitin ligase cereblon and induces the degradation of the transcription factors Ikaros and Aiolos, which are determinants of T-cell differentiation [296,297]. This drives T-cells towards an inflammatory Th1 phenotype, with increased secretion of IFN-γ [298,299]. While the combination of blinatumomab with lenalidomide was well tolerated and led to improved ORRs compared to blinatumomab alone [256,257], combinations of lenalidomide with checkpoint inhibitors yields unacceptable toxicities. Similarly, ibrutinib also drives skewing towards Th1 phenotypes via the inhibition of both BTK and IL-2 inducible T-cell kinase (ITK) [300,301]. However, when combined with nivolumab, only minimally improved response rates were seen in patients with CLL [302]. Likewise, idelalisib is a phosphoinositide 3-kinase (PI3K) inhibitor that is under investigation in combination with pembrolizumab in CLL and other B-NHLs (NCT02332980). Ultimately, it remains a considerable challenge to identify combination regimens that are appropriate for various lymphoma subtypes.

## 6. Conclusions

Immunotherapies have revolutionized the treatment of solid and hematologic malignancies alike. Clinical experience with bispecific antibodies and immune checkpoint modulators has begun to delineate which lymphoma subtypes are most amenable to such therapy, with an increased emphasis on the role of the TME in driving treatment sensitivities. Inflamed lymphomas, such as cHL, PMBCL, PCNSL, and PTL, are highly responsive to ICI therapy due, in part, to robust T-cell infiltrates, high PD-1/PD-L1 expression, and relatively high TMBs. As the role of the innate immune system in the TME becomes increasingly clarified, modulators of NK, TAM, and MDSC interactions have also entered development. However, numerous challenges remain, including the identification of suitable biomarkers and combination regimens, and future work will need to address these barriers in order to continue improving patient outcomes in lymphoma.

## Figures and Tables

**Table 1:** Clinical trials of immune checkpoint inhibitors in B-cell non-Hodgkin lymphoma.

Trial ID (Name)	Authors	Year	Intervention(s)	Phase	Disease(s)	N	CR	OR	PFS
NCT00089076	Ansell et al.	2009	Ipilimumab	I	FL DLBCL MCL	14 3 1	0% 33% 0%	7% 33% 0%	N.A.
NCT00904722	Westin et al.	2014	Pidilizumab + Rituximab	II	FL	29	52%	66%	N.A.
NCT01592370 (CHECKMATE-039)	Ansell et al.	2016	Nivolumab + Ipilimumab	Ib	B-NHL	15	0%	20%	median 1.5 months
NCT01592370 (CHECKMATE-039)	Lesokhin et al.	2016	Nivolumab	Ib	FL DLBCL Other B-NHL	10 11 10	10% 18% 0%	40% 36% 0%	median 23 weeks
-	Nayak et al.	2017	Nivolumab	I	PCNSL PTL	4 1	75% 100%	100% 100%	N.A
NCT01953692 (KEYNOTE-013)	Zinzani et al.	2017	Pembrolizumab	Ib	PMBCL	17	12%	41%	N.A.
NCT02220842	Palomba et al.	2017	Atezolizumab + Obinutuzumab	Ib	FL DLBCL	26 23	N.A. N.A.	57% 16%	N.A.
NCT02332980	Ding et al.	2017	Pembrolizumab	II	CLL CLL with RT	16 9	0% 11%	0% 44%	median 2.4 months median 5.4 months
NCT02446457	Nastoupil et al.	2017	Pembrolizumab + Rituximab	II	FL	25	60%	80%	N.A.
NCT02596971	Younes et al.	2017	Atezolizumab + Obinutuzumab + Bendamustine	Ib/II	untreated FL	15	67%	80%	N.A.
NCT02596971	Younes et al.	2018	Atezolizumab + R-CHOP	I/II	untreated DLBCL	40	78%	88%	N.A.
NCT01729806	Tuscano et al.	2019	Ipilimumab + Rituximab	I	FL Other B-NHL	13 20	15% 0%	38% 3%	median 2.6 months
NCT01953692 (KEYNOTE-013)	Armand et al.	2019	Pembrolizumab	Ib	PMBCL	21	33%	48%	median 10.4 months
NCT02038933 (CHECKMATE-139)	Ansell et al.	2019	Nivolumab	II	DLBCL s/p failed auto-HSCT DLBCL ineligible for auto-HSCT	87 34	3% 0%	10% 3%	median 1.9 months median 1.4 months
NCT02329847	Younes et al.	2019	Nivolumab + Ibrutinib	I/II	CLL/SLL FL DLBCL CLL with RT	36 40 45 20	0% 10% 16% 10%	61% 33% 36% 65%	N.A. median 9.1 months median 2.6 months median 5.0 months
NCT02576990 (KEYNOTE-170)	Armand et al.	2019	Pembrolizumab	II	PMBCL	53	13%	45%	median 5.5 months
NCT02581631 (CHECKMATE-436)	Zinzani et al.	2019	Nivolumab + BV	I/II	PMBCL	30	37%	73%	63.5% at 6 months
NCT02733042 (FUSION NHL-001)	Casulo et al.	2019	DurvalumabDurvalumab + Lenalidomide ± RituximabDurvalumab + Rituximab ± Bendamustine	I/II	DLBCL FL	38 22	8% 27%	18% 59%	median 2.5 months median 10.6 months
-	Smith et al.	2020	Pembrolizumab + R-CHOP	II	untreated DLBCL untreated FL	27 3	77%	90%	83% at 24 months
NCT02401048	Herrera et al.	2020	Durvalumab + Ibrutinib	Ib/II	FL DLBCL (GC) DLBCL (non-GC)	27 16 16	4% 6% 31%	26% 13% 38%	median 10.2 months median 2.9 months median 4.1 months
NCT02038946 (CHECKMATE-140)	Armand et al.	2021	Nivolumab	II	FL	92	1%	4%	median 2.2 months

All disease groups are relapsed/refractory unless otherwise specified. Abbreviations: N, number of patients; CR, complete response, OR, overall response; PFS, progression free survival; N.A., not assessed; s/p, status post; GC, germinal center; RT, Richter transformation.

**Table 2:** Clinical trials of immune checkpoint inhibitors in Hodgkin lymphoma.

Trial ID (Name)	Authors	Year	Intervention(s)	Phase	Disease(s)	N	CR	OR	PFS
NCT01592370	Ansell et al.	2015	Nivolumab	I	cHL	23	17%	87%	86% at 24 weeks
NCT01592370 (CHECKMATE-039)	Ansell et al.	2016	Nivolumab + Ipilimumab	Ib	cHL	31	19%	74%	N.A.
NCT01953692 (KEYNOTE-013)	Armand et al.	2016	Pembrolizumab	Ib	cHL	31	16%	65%	46% at 52 weeks
NCT02181738 (CHECKMATE-205)	Younes et al.	2016	Nivolumab	II	cHL	80	9%	66%	77% at 6 months
JapicCPI-142755	Maruyama et al.	2017	Nivolumab	II	cHL	16	25%	81%	60% at 6 months
NCT02453594 (KEYNOTE-087)	Chen et al.	2017	Pembrolizumab	II	cHL	210	22%	69%	72% at 6 months
NCT02181738 (CHECKMATE-205)	Armand et al.	2018	Nivolumab	II	cHL	243	16%	69%	median 14.7 months
NCT02572167	Herrera et al.	2018	Nivolumab + BV	I/II	cHL	61	61%	82%	89% at 6 months
NCT03114683 (ORIENT-1)	Shi et al.	2019	Sintilimab	II	cHL	92	N.A.	80%	N.A.
NCT02181738 (CHECKMATE-205)	Ramchandren et al.	2019	Nivolumab + AVD	II	untreated cHL	51	80%	84%	92% at 9 months
NCT02453594 (KEYNOTE-087)	Chen et al.	2019	Pembrolizumab	II	cHL	210	28%	72%	median 13.7 months
NCT03155425	Song et al.	2019	Camrelizumab	II	cHL	75	28%	76%	median 11.3 months
JapicCPI-142755	Maruyama et al.	2020	Nivolumab	II	cHL	16	31%	88%	median 11.7 months
NCT01896999	Diefenbach et al.	2020	Ipilimumab + BV Nivolumab + BV Ipilimumab + Nivolumab + BV	I/II	cHL	23 19 22	57% 61% 73%	76% 89% 82%	61% at 12 months 70% at 12 months 80% at 12 months
NCT02304458 (ADVL1412)	Davis et al.	2020	Nivolumab	I/II	cHL	12	10%	30%	N.A.
NCT02332668 (KEYNOTE-051)	Geoerger et al.	2020	Pembrolizumab	I/II	cHL	18	13%	60%	median 12.2 months
NCT02541604 (iMATRIX)	Geoerger et al.	2020	Atezolizumab	I/II	cHL	9	0%	22%	median 1.3 months ^a^
NCT02758717 (ACCRU)	Cheson et al.	2020	Nivolumab + BV	II	untreated cHL	46	48%	61%	median 18.3 months
NCT03004833 (NIVAHL)	Brockelmann et al.	2020	Nivolumab then AVD Nivolumab with AVD	II	untreated cHL	50 51	84% 83%	98% 100%	98% at 12 months 100% at 12 months
NCT03209973	Song et al.	2020	Tislelizumab	II	cHL	70	63%	87%	75% at 9 months

All disease groups are relapsed/refractory unless otherwise specified. ^a^ PFS reported for all diseases evaluated in trial without cHL subgroup analysis. Abbreviations: N, number of patients; CR, complete response, OR, overall response; PFS, progression free survival; N.A., not assessed.

**Table 3:** Clinical data of immune checkpoint inhibitors in T-cell lymphoma.

Trial ID (Name)	Authors	Year	Intervention(s)	Phase	Disease(s)	N	CR	OR	PFS
NCT01592370 (CHECKMATE-039)	Lesokhin et al.	2016	Nivolumab	Ib	MF PTCL Other CTCL Other non-CTCL	13 5 3 2	0% 0% 0% 0%	15% 40% 0% 0%	median 10 weeks
NCT01592370 (CHECKMATE-039)	Ansell et al.	2016	Nivolumab + Ipilimumab	Ib	T-NHL	11	0%	9%	median 2.0 months
-	Kwong et al.	2017	Pembrolizumab	R ^a^	NKTCL	7	71%	100%	N.A.
-	Li et al.	2018	Pembrolizumab	R ^a^	NKTCL	7	29%	57%	median 4.8 months
NCT03075553	Bennani et al.	2019	Nivolumab	II	ALK-neg ALCL AITL PTCL Other Non-CTCL	1 6 3 2	100% 17% 0% 0%	100% 17% 33% 50%	median 1.9 months
NCT02535247	Barta et al.	2019	Pembrolizumab	II	PTCL FTL MF Other non-CTCL	7 4 3 3	0% 50% 33% 33%	14% 50% 33% 33%	median 3.2 months
NCT02243579 (CITN-10)	Khodadoust et al.	2020	Pembrolizumab	II	MF SS	9 15	0% 13%	56% 27%	65% at 1 year

All disease groups are relapsed/refractory unless otherwise specified. ^a^ Retrospective case series analyses. Abbreviations: N, number of patients; CR, complete response, OR, overall response; PFS, progression free survival; N.A., not assessed.

**Table 4:** Clinical trials of immune checkpoint inhibitors after autologous hematopoietic stem cell transplantation (HSCT).

Trial ID (Name)	Authors	Year	Intervention(s)	Phase	Disease(s)	N	CR	OR	PFS
NCT00060372 (CTEP 6082)	Bashey et al.	2009	Ipilimumab after allo-HSCT	I	cHL B-NHL	14 3	14% 0%	14% 33%	N.A.
NCT00532259	Armand et al.	2013	Pidilizumab after auto-HSCT	II	DLBCL	35	34%	51%	72% at 16 months
NCT01822509	Davids et al.	2016	Ipilimumab after allo-HSCT	I/Ib	cHL B-NHL	7 4	0% 0%	14% 0%	N.A.
NCT01919619	Khouri et al.	2018	Ipilimumab + Lenalidomide after HSCT	II	B-NHL (allo-HSCT) B-NHL (auto-HSCT)	8 6	38% 83%	75% 83%	56% at 12 months 86% at 12 months
NCT02362997	Frigault et al.	2020	Pembrolizumab after auto-HSCT	II	DLBCL	29	59%	59%	58% at 18.5 months

All disease groups are relapsed/refractory unless otherwise specified. Abbreviations: N, number of patients; CR, complete response, OR, overall response; PFS, progression free survival; N.A., not assessed.

**Table 5:** Listed trials of investigational checkpoint inhibitors in lymphoma.

Trial ID	Phase	Target(s)	Intervention(s)	Population(s)	Status
NCT03489343	I	TIM-3	Sym023	Advanced malignancies, including lymphomas	Completed
NCT03311412	I	TIM-3 LAG-3	Sym021 + Sym022 (LAG-3) Sym021 + Sym023 (TIM-3) Sym021 + Sym022 + Sym023	Advanced malignancies, including lymphomas	Recruiting
NCT02061761	I	LAG-3	BMS-986016 ± Nivolumab (PD-1)	Hematologic malignancies, including cHL, NHL, CLL, and MM	Active
NCT03538028	I	LAG-3	INCAGN02385	Advanced malignancies, including DLBCL	Completed
NCT03365791	II	LAG-3	LAG525 + PDR001 (PD-1)	Advanced malignancies, including DLBCL	Completed
NCT03005782	I	LAG-3	REGN3767 ± Cemiplimab (PD-1)	Advanced solid malignancies or lymphomas	Recruiting
NCT03489369	I	LAG-3	Sym022	Advanced malignancies, including lymphomas	Completed
NCT04254107	I	TIGIT	SGN-TGT ± Pembrolizumab	Advanced malignancies, including cHL, DLBCL, and PTCL	Recruiting
NCT04353830	I	TIGIT	IBI939 ± Sintilimab (PD-1)	Advanced malignancies (no further specification)	Recruiting

**Table 6:** Clinical trials of innate checkpoint inhibitors in lymphoma.

Trial ID	Authors	Year	Intervention(s)	Target(s)	Phase	Disease(s)	N	CR	OR	PFS
NCT02953509	Advani et al.	2018	Hu5F9-G4	CD47	Ib	DLBCL FL	15 7	33% 43%	40% 71%	91% at 6.2 months 91% at 8.1 months
NCT02216409	Sikic et al.	2019	Hu5F9-G4	CD47	I	DLBCL	2	0%	50%	N.A.
NCT02367196	Abrisqueta et al.	2019	CC-90002	CD47	I	B-NHL	24	4%	13%	N.A.
NCT02663518	Johnson et al.	2019	TTI-621	CD47	Ia	CTCL with SS	5	0%	80%	N.A.
NCT03530683	Patel et al.	2020	TTI-622	CD47	I	cHL B-NHL T-NHL	5 16 4	0% 6% 0%	0% 19% 50%	N.A.
NCT02663518	Ansell et al.	2021	TTI-621 TTI-621 + Rituximab TTI-621 + Nivolumab	CD47	I	B-NHL (monotherapy) B-NHL (w/Rituximab) cHL (monotherapy) cHL (w/Nivolumab) T-NHL CLL	21 35 20 4 40 3	5% 9% 0% 25% 3% 0%	10% 23% 5% 50% 20% 0%	N.A
EUDRACT 2009-011526-33	Vey et al.	2018	Lirilumab	KIR	I	CLL other B-NHL	6 11	0%	0%	median 19.6 months median not reached
NCT01592370	Armand et al.	2020	Lirilumab + Nivolumab	KIR	Ib	cHL B-NHL T-NHL	21 32 9	24% 3% 0%	76% 13% 22%	62% at 12 months median 1 months median 6 months

All disease groups are relapsed/refractory unless otherwise specified. Abbreviations: N, number of patients; CR, complete response, OR, overall response; PFS, progression free survival; N.A., not assessed.

**Table 7:** Clinical trials of immune checkpoint stimulators in lymphoma.

Trial ID (Name)	Authors	Year	Intervention(s)	Target(s)	Phase	Disease(s)	N	CR	OR	PFS
NCT01471210 (CA186-011)	Segal et al.	2017	Urelumab	CD137 (4-1BB)	I	B-NHL	11	N.A.	N.A.	N.A.
NCT01307267	Segal et al.	2018	Utomilumab	CD137 (4-1BB)	I	Lymphoma ^a^	2	N.A.	N.A.	N.A.
NCT01307267	Gopal et al.	2020	Utomilumab + Rituximab	CD137 (4-1BB)	I	FL DLBCL MCL CLL/SLL MZL NLPHL	47 7 6 3 2 1	9% 0% 0% 0% 0% 0%	23% 14% 17% 0% 0% 100%	median 4.6 months ^b^
NCT01471210 (CA186-011)	Timmerman et al.	2020	Urelumab	CD137 (4-1BB)	I	DLBCL FL other B-NHL	31 29 12	0% 6% 17%	6% 12% 17%	median 8.1 weeks median 8.9 weeks median 13.4 weeks
NCT01775631 (CA186-017)	Timmerman et al.	2020	Urelumab + Rituximab	CD137 (4-1BB)	Ib	DLBCL FL	29 17	7% 12%	10% 35%	median 9.0 weeks median 40.4 weeks
NCT01470134	Ansell et al.	2020	Varlilumab	CD27	I	DLBCL FL other B-NHL cHL PTCL CTCL MF	10 6 2 11 2 1 2	0% 0% 0% 9% 0% 0% 0%	0% 0% 0% 9% 0% 0% 0%	N.A.
NCT00103779	Advani et al.	2009	Dacetuzumab	CD40	I	DLBCL FL MCL MZL CLL/SLL other NHL	21 12 10 3 1 3	5% 0% 0% 0% 0% 0%	10% 0% 10% 33% 0% 0%	N.A.
NCT00283101	Furman et al.	2010	Dacetuzumab	CD40	I	CLL	12	0%	0%	N.A.
NCT00108108	Byrd et al.	2012	Lucatumumab	CD40	I	CLL	24	0%	4%	N.A.
NCT00655837	Forero-Torres et al.	2013	Dacetuzumab + Rituximab + Gemcitabine	CD40	Ib	DLBCL	30	20%	47%	median 25 weeks
NCT00435916	de Vos et al.	2014	Dacetuzumab	CD40	II	DLBCL FL MZL	40 3 2	5% 0% 0%	8% 33% 0%	median 36 days
NCT00670592	Fanale et al.	2014	Lucatumumab	CD40	Ia/II	FL DLBCL MALT MCL cHL	21 34 7 12 37	5% 6% 14% 0% 0%	33% 12% 43% 0% 14%	N.A.
NCT00529503	Fayad et al.	2015	Dacetuzumab + R-ICE Placebo + R-ICE	CD40	IIb	DLBCL	75 76	33% 36%	67% 64%	median 12.1 months median 6.5 months

All disease groups are relapsed/refractory unless otherwise specified. ^a^ Subtype not specified. ^b^ For patients with NHL. Abbreviations: N, number of patients; CR, complete response, OR, overall response; PFS, progression free survival; N.A., not assessed.

**Table 8:** Clinical trials of bispecific T-cell-engaging constructs in lymphoma.

Trial ID (Name)	Authors	Year	Intervention(s)	Target(s)	Format	Phase	Disease(s)	N	CR	OR	PFS
NCT00274742	Goebeler et al.	2016	Blinatumomab	CD3 × CD19	BiTE	I	FL MCL DLBCL	15 7 11	40% 43% 36%	80% 71% 55%	N.A.
NCT01741792	Viardot et al.	2016	Blinatumomab	CD3 × CD19	BiTE	II	DLBCL	21	19%	43%	median 3.7 months
NCT02910063	Coyle et al.	2020	Blinatumomab	CD3 × CD19	BiTE	II	B-NHL	41	22%	37%	median 8.4 months ^a^
NCT02568553	Poh et al.	2019	Blinatumomab + Lenalidomide	CD3 × CD19	BiTE	I	B-NHL	18	50%	83%	median 8.3 months
NCT03023878	Katz et al.	2019	Blinatumomab after R-chemotherapy	CD3 × CD19	BiTE	II	DLBCL	28	N.A.	89%	N.A.
NCT03931642 (BLINART)	Guieze et al.	2020	Blinatumomab after R-CHOP	CD3 × CD19	BiTE	II	CLL with RT	5	40%	60%	N.A.
NCT03075696 (NP30179)	Morschhauser et al.	2019	Glofitamab and Obinutuzumab	CD3 × CD20	BsAb	I/Ib	B-NHL (all patients) B-NHL (highest dose)	21 10	43% 80%	48% 90%	N.A.
NCT03075696 (NP30179)	Hutchings et al.	2020	Glofitamab and Obinutuzumab	CD3 × CD20	BsAb	I/Ib	aggressive B-NHL indolent B-NHL	24 8	29% 75%	50% 100%	N.A.
NCT02290951	Bannerji et al.	2018	Odronextamab (REGN1979)	CD3 × CD20	BsAb	I	DLBCL FL MCL	15 7 2	0% 71% 0%	40% 100% 100%	N.A.
NCT02290951	Bannerji et al.	2020	Odronextamab (REGN1979)	CD3 × CD20	BsAb	I	FL (≥5 mg) FL (≥80 mg) DLBCL (no prior CAR, ≥5 mg) DLBCL (no prior CAR, ≥80 mg) DLBCL (relapse after CAR, ≥5 mg) DLBCL (relapse after CAR, ≥80 mg)	28 16 30 10 23 21	75% 69% 30% 60% 22% 24%	93% 94% 47% 60% 30% 33%	median 12.8 months median 12.8 months median 5.1 months median 11.1 months median 2.5 months median 2.5 months
NCT03625037	Hutchings et al.	2020	Epcoritamab (GEN3013, SQ)	CD3 × CD20	BsAb	I/II	DLBCL FL	18 8	33% 25%	67% 100%	N.A.
NCT02500407 (GO29781)	Schuster et al.	2019	Mosunetuzumab (RG7828)	CD3 × CD20	BsAb	I/Ib	aggressive B-NHL indolent B-NHL	119 64	19% 42%	35% 64%	N.A.
NCT02500407 (GO29781)	Assouline et al.	2020	Mosunetuzumab (RG7828)	CD3 × CD20	BsAb	I/Ib	FL	62	50%	68%	median 11.8 months
NCT02500407 (GO29781)	Matasar et al.	2020	Mosunetuzumab (RG7828, SQ)	CD3 × CD20	BsAb	I/Ib	aggressive B-NHL indolent B-NHL	15 7	20% 29%	60% 86%	N.A.
NCT03677154 (GO40554)	Olszewski et al.	2020	Mosunetuzumab (RG7828)	CD3 × CD20	BsAb	I/II	untreated DLBCL	19	42%	58%	N.A.
NCT03677141 (GO40515)	Phillips et al.	2020	Mosunetuzumab (RG7828) + CHOP	CD3 × CD20	BsAb	I/II	B-NHL untreated DLBCL	7 36	71% 85%	86% 96%	N.A.

All disease groups are relapsed/refractory unless otherwise specified. ^a^ For patients who achieved CR. Abbreviations: N, number of patients; CR, complete response, OR, overall response; PFS, progression free survival; N.A., not assessed.

**Table 9:** Clinical trials of bispecific NK-cell-engaging constructs in lymphoma.

Trial ID	Authors	Year	Intervention(s)	Target(s)	Format	Phase	Disease(s)	N	CR	OR	PFS
NCT01221571	Rothe et al.	2015	AFM13	CD16 × CD30	TandAb	I	cHL	26	0%	12%	N.A.
NCT02321592	Sasse et al.	2020	AFM13	CD16 × CD30	TandAb	II	cHL	24	4%	17%	12.6% at 12 months ^a^
NCT02665650	Bartlett et al.	2020	AFM13 + Pembrolizumab	CD16 × CD30	TandAb	Ib	cHL	30	37%	83%	77% at 6 months ^b^

All disease groups are relapsed/refractory unless otherwise specified. ^a^ Terminated early due to low recruitment. ^b^ Interim analysis. Abbreviations: N, number of patients; CR, complete response, OR, overall response; PFS, progression free survival; N.A., not assessed.
